# Does sleep help children to generalise features like adults?

**DOI:** 10.1111/jsr.14432

**Published:** 2024-12-08

**Authors:** Eva‐Maria Kurz, Clara Marie Schreiber, Konstantin Kölle, Zeynep Tunçel, Paula Theresa Meyer, Hong‐Viet V. Ngo‐Dehning, Annette Conzelmann, Alexander Prehn‐Kristensen

**Affiliations:** ^1^ Institute of Medical Psychology and Behavioural Neurobiology University of Tübingen Tübingen Germany; ^2^ Department of Child and Adolescent Psychiatry, Psychosomatics and Psychotherapy University Hospital of Psychiatry and Psychotherapy, University of Tübingen Tübingen Germany; ^3^ Institute of Child and Adolescent Psychiatry and Psychotherapy, Centre for Integrative Psychiatry, School of Medicine University Medical Centre Schleswig‐Holstein‐ Campus Kiel Kiel Germany; ^4^ Department of Psychology University of Essex Colchester UK; ^5^ German Center for Mental Health (DZPG), Partner Site Tübingen Tübingen Germany; ^6^ Department of Psychology (Clinical Psychology II) PFH – Private University of Applied Sciences Göttingen Germany; ^7^ Department of Psychology, Faculty of Human Sciences MSH Medical School Hamburg – University of Applied Sciences and Medical University Hamburg Germany

**Keywords:** children, development, emotion, generalisation, gist, memory, sleep, social

## Abstract

Children and adults have been shown to benefit from sleep with regard to the consolidation of declarative memories. Especially during childhood, the generalisation of information from social and non‐social contexts is important for adaptable behaviour in new situations and might show specific features in children. Here, we investigated whether adults (*n* = 18) and children (*n* = 19) differ in their generalisation of features assessed in wake and sleep conditions. In a social paradigm, certain face features were associated with different types of offers (fair, unfair, friendly). While children tended to better recognise these faces, adults were better than children at associating the type of offer to unknown faces sharing these features with the previously encoded faces in the sleep condition. To assess generalisation of features in a non‐social context, a probabilistic evaluative conditioning paradigm was used, where stimuli were associated with positive or negative values. We found no difference between children and adults or between the sleep and wake condition in the change in evaluation of the conditioned stimuli when paired congruently with a predefined value (positive/negative). Together, our results suggest a differential feature generalisation from mainly social contexts in children compared with adults.

## 1 INTRODUCTION

Both in children and adults, sleep has been shown to support the consolidation of declarative memories (Hoedlmoser et al., 2022). According to the active systems consolidation framework, during sleep, the repeated parallel reactivation of previously encoded memory traces in the neocortex and the hippocampus fosters the transmission of the more context‐dependent episodic hippocampal information into neocortical long‐term stores (Brodt et al., 2023; Klinzing et al., 2019). In addition to a strengthening of memory representations, this process has also been proposed to lead to a qualitative change in memories, such as the formation of schema memories (Klinzing et al., 2019). Pre‐existing schemas, which are highly complex knowledge structures, might influence the abstraction of rules or the gist information that is extracted, with the repeated extraction of gist information possibly contributing to the abstraction of concepts (Gilboa & Marlatte, 2017). The formation of schemas and rules is especially important during development, when children accumulate vast amounts of new information, which has to be used to either form new concepts or to integrate it into existing knowledge structures. Since schemas are used to guide flexible behaviour in new, yet similar situations, it is an integral part of human cognitive development to form "appropriate" and flexible schemas (see Hartley et al., 2021 for a review on adaptive learning and memory across development).

The brain structures involved in memory consolidation and schema networks, that is, the prefrontal cortex and hippocampus, undergo protracted maturational processes (Gogtay et al., 2004; Lee et al., 2017). At the same time, sleep architecture changes drastically during the first two decades of life (Scholle et al., 2011). Changes during childhood mainly apply to non‐REM sleep, with its hallmarks (e.g. duration of slow wave sleep [SWS], density of spindles and slow oscillations; [Ohayon et al., 2004]) being repeatedly associated with the retention of declarative memories in children (e.g. Hoedlmoser et al., 2014; Lokhandwala & Spencer, 2021) and adults (Marshall et al., 2006; Ngo et al., 2013). Indeed, there are studies showing an advantage in sleep‐associated consolidation of declarative memory content in children (aged between 7 and 12 years) compared with adults with respect to neutral (Peiffer et al., 2020), emotional (Prehn‐Kristensen et al., 2013), or rewarded information (Prehn‐Kristensen et al., 2018). Moreover, it has been hypothesised that the increased SWS might enable children to acquire high amounts of memory representations and to establish cognitive schemas (Wang et al., 2018). However, there is only one study directly comparing children (8–11 years) and adults regarding the influence of sleep in the transformation of memories (Wilhelm et al., 2013). In that study, participants implicitly learned the sequence of a serial reaction time task prior to sleep or wakefulness. When asked to explicitly recall the underlying sequence, children showed greater explicit knowledge than adults in the sleep condition, pointing towards a greater benefit of sleep in the reorganisation and transformation of memories in children (Wilhelm et al., 2013). To date, it is unclear whether children similarly show a greater sleep‐benefit than adults in the generalisation of features to new, yet similar stimuli that have been extracted incidentally during a reward‐based task in a social context and an evaluative conditioning paradigm.

To this end, a group of children between 8 to 12 and adults between 20 to 30 years each participated in a sleep and wake condition, performing self‐developed tasks to abstract features in a social and non‐social context. Due to the within‐subject design of our study, feature abstraction was only implicitly assessed by generalisation. We hypothesised that nighttime sleep more than daytime wakefulness benefits the preferential extraction of features relevant for the evaluation of unknown stimuli. Furthermore, we expected children to show a greater sleep‐benefit than adults.

## 2 METHODS

### 2.1 Participants

Eighteen adults between 20 and 30 years (9 female, *M* = 25 years, SD = 2.59) and 19 children between 8 and 12 years (12 female, *M* = 10.47 years, *SD* = 1.57) participated in the study. Inclusion criteria were IQ > 80 and no severe sleep problems (see Table S1). One adult participant had to be excluded due to napping between encoding and retrieval in the wake condition. From the final sample, all participants had an IQ > 83 as assessed with the CFT 20‐R (Weiß, 2008) or the CFT 1‐R (Weiß & Osterland, 2013) when the participants were younger than 8.5 years (adults: *M* = 109, *SD* = 15.3, children: *M* = 103, SD = 16, group comparison: *t*(34) = −1.17, *p* = 0.25).

Children and adults were recruited through flyers distributed at public spaces (e.g. public library) and personal contacts. The study was approved by the ethics committee of the University of Kiel. All participants gave written informed consent and received 70€ as monetary compensation.

### 2.2 Procedure

Participants were informed about the procedure and objectives of the study via a first telephone call. All further experimental sessions took place in the participants' home. At the first session, participants gave written informed consent. Participants and, if applicable their parents, filled out questionnaires assessing sleep quality and behaviour. For children, sleep quality was assessed using two different questionnaires: Parents reported on their child's sleep using the Children's Sleep Habit Questionnaire (CSHQ‐DE, Schlarb et al., 2010), while children filled out the Sleep‐Self‐Report (SSR‐DE, Schwerdtle et al., 2010). The adult sample provided information on sleep quality filling out the Pittsburgh Sleep Quality Index (PSQI, Buysse et al., 1989). For results and additional participant characteristics see Table S1.

All children and adults participated in a sleep and wake condition, with an interval of 2 weeks in between and the order of conditions being almost balanced across participants (children: sleep first *n* = 10 and wake first *n* = 9; adults: sleep first *n* = 9, wake first *n* = 8). Each condition comprised an encoding and retrieval session with a 12‐hour retention interval between the two sessions. Encoding in the wake condition started at 8 a.m. and retrieval at 8 p.m., with the instruction not to take a nap during the retention interval. In the sleep condition, encoding took place at 8 pm and retrieval 12 h later in the next morning.

At the beginning of each encoding and retrieval session, participants performed three control tasks, to control for current alertness, mood, and tiredness, and thereafter two memory tasks. The order of the two memory tasks within a condition was balanced across participants. Due to the within‐design of the study, two parallel versions of each memory task were created, with their order being almost balanced across sleep and wake condition. In the encoding session, each memory task comprised an encoding and a baseline test and lasted 1 hour. In the retrieval session, participants performed the memory task (delayed) a second time (approximately 30 min).

To ensure minimal effort for the participants, that is, keeping the number of sessions to a minimum, intelligence testing was done after the retrieval session of the very last experimental day.

### 2.3 Reward‐based feature generalisation in a social context

**FIGURE 1:**
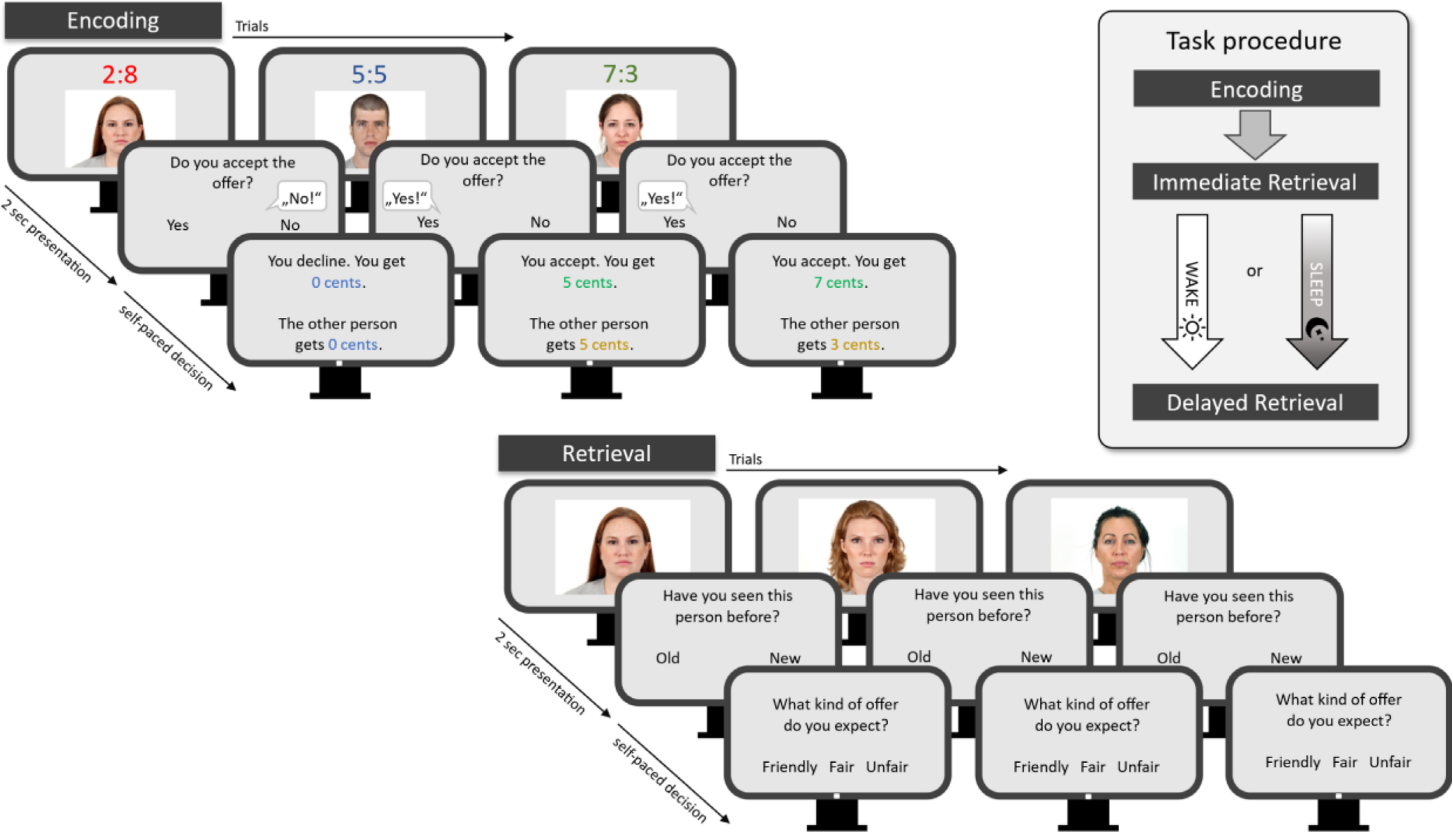
Reward‐based feature generalisation in a social context – Procedure. For detailed explanations, see Methods.

This task was based on the ultimatum game (Güth et al., 1982). Since a within‐study design was employed two sets of pictures were used (Version A and B) with all pictures taken from the Chicago Face Database (Ma et al., 2015, see also Supporting Information "Version balancing"). Stimuli in the encoding phase consisted of 45 different faces which were each paired with one of three possible consequences, that is, a fair, unfair or friendly split of money, with 15 trials each. In the fair offer, 10 cents (Euro) were split equally between the proposer (face presented on the screen) and the responder (participant); in the unfair offer, the 10 cents were split 8:2 in favour of the proposer; and in the friendly offer, the participant received 7 cents, while 3 cents stayed with the proposer (Figure 1). For each proposal the participant had to decide whether to accept the offer (yes/no response), with a "yes" response indicating that both proposer and responder received the money from the accepted offer and a "no" response that no one received the money. After the task, the participants received the money from all accepted offers. The participants knew that they would have to recognise the faces of the proposers later.

Unbeknownst to the participants, the proposers' faces within one consequence shared some features, e.g. faces offering a fair money split mostly had short to medium‐length hair or red hair, while those belonging to the unfair consequence condition mostly had bright or long open hair. While in version A the combination of hair colour and the way the hair was worn was the key information, it was the colour of the eyes, sex, and hairstyle (curly or straight) in version B.

Immediately after encoding a first recognition task (baseline recognition) started, comprising 30 trials. Fifteen of the previously presented faces were intermixed with 15 unknown faces, whose characteristics fulfilled one of the three consequences from the encoding session. For each face, the participants had to indicate whether they had seen it before (old/new response) and whether the presented person would make a fair, unfair, or friendly offer. Since task instructions for the whole task were presented right before encoding, the participants were aware that there are three types of offers (fair, unfair, or friendly).

In the retrieval session, after the 12‐hour retention interval, the participants performed the recognition task a second time (delayed recognition and offer prediction). The task consisted of 60 trials, including 30 known faces which had not been part of the baseline recognition task, and another 30 unknown distractors.

Recognition memory for faces, offer recognition (for old faces), and offer prediction accuracy (for new faces), were analysed by means of d‐prime (z(hit rate)‐z(false alarm rate)). Values of 0 and 1 for hit and false alarm rates were replaced by adding 0.5 to the number of hits or false alarms divided by the number of signal and noise trials plus 1, respectively (Hautus, 1995). To control for initial levels of encoding, the difference between the baseline and delayed retrieval test was calculated.

### 2.4 Evaluative conditioning in a non‐social context

**FIGURE 2:**
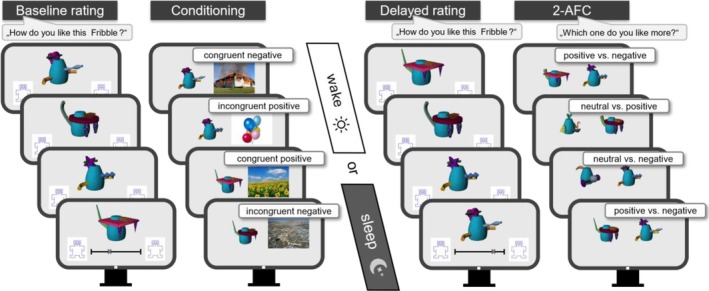
Evaluative Conditioning Task – Procedure. For detailed explanations, see Methods and Supplement; 2‐AFC, two‐alternative forced choice.

The task is based on paradigms investigating the generalisability of evaluative conditioning to new objects sharing the "value" with the originally paired neutral stimulus (Hütter et al., 2014). As neutral stimuli, the so‐called Fribbles were used (Williams & Simons, 2000; Stimulus images courtesy of Michael J. Tarr, Carnegie Mellon University, http://www.tarrlab.org/). Fribbles are completely unique constructs (Figure 2 and S1) that participants have no prior experience with, thus making them suitable for representing neutral stimuli (Barry et al., 2014). As with the social gist abstraction paradigm, two parallel versions were used (A and B). For each version, eight Fribbles were derived from two different Fribble species (four Fribbles each, see Table S2): while both species share the same base body, they each have species‐unique appendages that varied within a species. Unconditioned stimuli (UCS) pictures from the International Affective Picture System (IAPS, Lang et al., 1997) and the Mnemonic Similarity Task (Kirwan et al., 2007; Stark et al., 2013) were used, which were chosen based on experiences made in previous studies (e.g. Prehn‐Kristensen et al., 2009, 2013). To assess generalisation based on the concept of evaluative conditioning, a probabilistic allocation of Fribbles (conditioned stimuli, CS) to the UCS was used. Four different experimental steps were carried out. The first two steps were part of the encoding session while the last two steps were part of the retrieval session.

#### 2.4.1 Baseline evaluation

Encoding began with an evaluation of the initially neutral Fribbles. Here, participants had to rate how much they liked each of all eight unknown Fribbles using a visual analogue scale (VAS), ranging from 0 to 100 based on the valence scale of the self‐assessment manikin (SAM, Bradley & Lang, 1994, see Figure 2).

#### 2.4.2 Evaluative conditioning

During the subsequent evaluative conditioning session participants were presented with the eight Fribbles along with one of eight emotionally charged pictures serving as UCS. The pictures differed with respect to their emotional content (negative or positive valence). Emotionally positively charged pictures were photographs of balloons, pizza, cake, a cookie, and a sunflower field; emotionally negatively charged pictures were photographs of a dump, a burning house, a flood, garbage on the beach, and exhaust fumes in an industrial area. The Fribbles in turn differed with respect to their appendages; however, unknown to the participants, specific appendages were critical for the allocation to the two valence categories. Since both species within a version shared the same base body but each had species‐unique appendages, allocation to the negative and positive valence category was based on these unique features. However, this allocation was not deterministic but probabilistic (Table S2). To enable this, in 120 evaluative conditioning trials, the eight Fribbles were presented along with the UCS in the following manner: Four of the eight Fribbles (two per species) were always paired with an emotional picture that matched the predefined valence category (congruent trials), while the other four Fribbles (two per species) were presented along with an emotional picture that did not match the predefined valence category (incongruent trials). Fribbles that were paired congruently were presented along the UCS 25 times each (100 trials in total), while Fribbles that were paired incongruently were presented along the UCS five times each (20 trials in total). This resulted in an 83% rate of congruent and a 17% rate of incongruent trials.

#### 2.4.3 Delayed evaluation

After 12 hours, during the delayed rating session participants evaluated the valence of the eight conditioned Fribbles again, to assess possible effects of evaluative conditioning using the same VAS as mentioned above. Thus, for congruently paired Fribbles we were able to analyse whether the baseline evaluation changed into the direction of the UCS, with Fribbles presented with negative UCS now showing a more negative rating and for those associated with positive UCS a more positive rating. Since in 17% of the presented trials, Fribbles were paired incongruently, the analyses of those data enabled us to assess both item and gist memory: For incongruently paired Fribbles, a change of evaluation in the direction of the category valence would indicate gist memory, whereas a change in evaluation in the direction of the actual co‐presented UCS would indicate item memory.

#### 2.4.4 Feature generalisation

Finally, in a two‐alternative forced‐choice task (2‐AFC), participants were presented 25 times with a set of two new Fribbles to rate which of both Fribbles subjectively appeared more positive ("Which one of these two Fribbles do you like more?"). Fribble pairs were composed of unknown Fribbles, however, (i) from known species (and therefore being similar to those who had been conditioned: either positive or negative), or (ii) from unknown species (sharing only the same base body with known species and therefore being dissimilar), with the latter always representing neutral stimuli. Here, Fribble pairs were presented in one of the following three possible combinations: "positive vs. neutral" (7×), "positive vs. negative" (11×), and "negative vs. neutral" (7×). For more details see Figures S2 and S3. Positive and negative refer to the predefined valence categories, a neutral Fribble matched neither the positive nor the negative valence category (i.e. having completely new appendages). Since the question was which Fribble the participant liked more, correct decisions would be "neutral" when compared with Fribbles belonging to the "negative" valence category, and "positive" when paired with Fribbles belonging to the "neutral" or "negative" species. For each participant, we calculated the percentage of correct decisions.

### 2.5 Control measures

All participants performed a subtest of a test battery assessing attention (Zimmermann et al., 2005) prior to encoding and retrieval to control for current levels of alertness. Furthermore, children and adults rated their current emotional state using the scales valence, arousal, and dominance (each rated on a 9‐point scale) of the SAM before and after performing the memory tasks at encoding and retrieval. Subjective tiredness was assessed using a VAS (from 0 = very tired to 100 = very awake) at the beginning of encoding and retrieval sessions. Analyses of control measures indicated no influence of alertness and sleepiness on memory performance. See supplemental information "control measures" for a detailed description of results.

### 2.6 Statistical analyses

Statistical analyses were done in R version 4.3.1 (R Core Team, 2023). Performance in both tasks were analysed using linear mixed‐effects models using the R package *lme4* (Bates et al., 2015). The library *lmerTest* (Kuznetsova et al., 2017) was used to obtain F‐statistics and *p*‐values based on Type III sum of squares and Satterthwaite's method. Post‐hoc pairwise comparisons were calculated using the *emmeans* package (Lenth, 2023). We report uncorrected and Bonferroni‐corrected (*p*_corr_) *p*‐values. The models for the reward‐based task included the fixed effects group (adult vs. child), condition (sleep vs. wake) and consequence (friendly vs. fair vs. unfair) and their interaction terms, as well as random intercepts for participants. Separate models were calculated for the following outcome measures, with each being the difference between d‐prime from the delayed and immediate retrieval: the recognition of faces; the recognition of the associated offer for known (old) faces and the prediction of offers for unknown (new) faces.

For the evaluative conditioning task, two separate models for congruently and incongruently paired Fribbles were calculated to inspect the change in evaluation. These models comprised the fixed effects evaluation time (baseline vs. after conditioning), group (adults vs. children), condition (sleep vs. wake), category valence (positive vs. negative), their interaction terms as well as random intercepts for participant. For the 2‐AFC task, the model comprised the fixed effects group (adults vs. children), condition (sleep vs. wake), comparison (positive/neutral vs. positive/negative vs. negative/neutral), as well as all possible interaction terms and random intercepts for participants.

## 3 RESULTS

### 3.1 Reward‐based feature generalisation in a social context

#### 3.1.1 Acceptance rate

First, to control for overall effects of manipulation during the encoding sessions, we investigated whether the acceptance rate of an offer was dependent on the consequence of the offer, the group, or the condition. As expected, unfair offers (*M* = 21%, *SEM* = 3.84) were less often accepted than fair and friendly offers (all *p*_corr_ < 0.001, main effect type of offer: *F*(2,180) = 274, *p* < 0.001). Possibly due to ceiling effects, there was no difference in acceptance rates between fair (*M* = 94%, *SEM* = 1.41) and friendly offers (*M* = 91%, *SEM* = 2.53, *t*(191) = −0.71, *p* > 0.479). Adults (*M* = 74%, *SEM* = 4.02) generally accepted more offers than children (*M* = 64%, *SEM* = 3.86, main effect age group: *F*(1,36) = 5.49, *p* = 0.025). However, there were neither differences between the sleep and wake condition (*F*(1,180) = 0.002, *p* = 0.96), nor any significant interactions (all *p* > 0.518).

#### 3.1.2 Recognition memory

**FIGURE 3:**
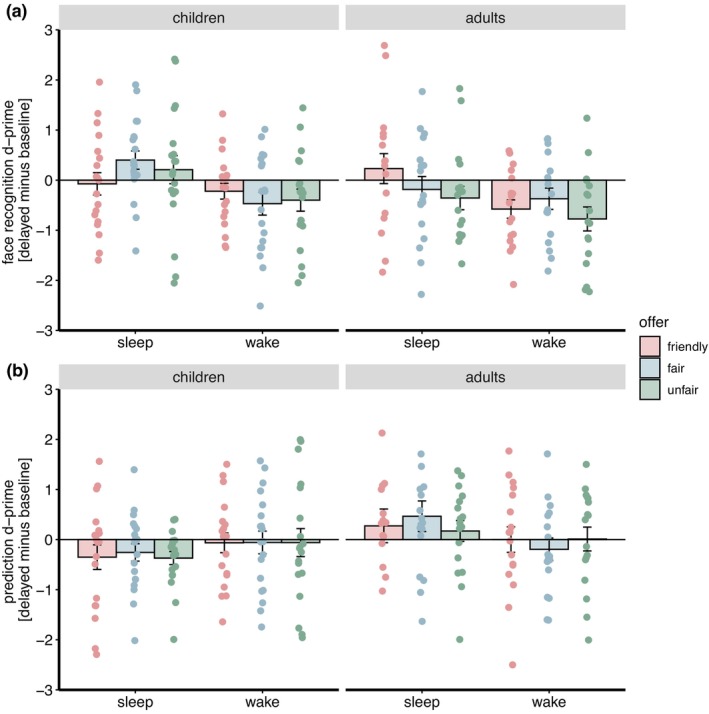
Reward‐based feature generalisation in a social context: Recognition performance (d‐prime) (a) and offer prediction performance for new faces (b) dependent on age group, condition, and type of offer as the difference between delayed and immediate retrieval.

As expected there was an overall benefit of sleep on face recognition: recognition performance remained stable across the interval filled with nighttime sleep (*M* = 0.06, *SEM* = 0.09), and deteriorated after daytime wakefulness (*M* = −0.42, *SEM* = 0.08, main effect condition: *F*(1,179.997) = 15.46, *p* < 0.001). Although the three‐way interaction between age group × condition × type of offer failed to reach significance (*F*(2,179.997) = 2.32, *p* = 0.101) we investigated the effects of condition for each combination of age group and type of offer. These six tests showed in adults a better recognition performance in the sleep condition compared with the wake condition for faces from the friendly offer category (*t*(191) = 2.42, *p* = 0.016, although *p*_corr_ = 0.098) and in children a sleep benefit only for faces from the fair offer category (*t*(191) = 2.76, *p* = 006, *p*_corr_ = 0.038, all other *p* > 0.055, Table S3). Further results of the face recognition memory (independent of sleep) were as follows: There was no difference in face recognition with respect to the type of offer (main effect type of offer: *F*(2,179.997) = 0.796, *p* = 0.453, Figure 3a). While there was a trend that children (*M* = −0.09, *SEM* = 0.09) showed less forgetting than adults (*M* = −0.34, *SEM* = 0.10, main effect group: *F*(1,35.997) = 3.67, *p* = 0.063), there was no interaction between age group and condition (*F*(1,179.997) = 0.08, *p* = 0.783) or between any of the other factors involved in two‐way interactions (all *p* > 0.371).

Next, we analysed whether participants were able to correctly recall the proposers' offers of old pictures. This, however, yielded no difference in condition (*F*(1,180) = 0.003, *p* = 0.955), group (*F*(1,180) = 0.36, *p* = 0.551), type of offer (*F*(2,180) = 0.317, *p* = 0.729) or their interaction (all *p* > 0.120, Table S4).

#### 3.1.3 Prediction rate

For unknown pictures, we analysed whether participants were able to correctly predict the associated offer based on the hidden rule. A significant condition × age group interaction (*F*(1,180) = 7.10, *p* = 0.008) did not show differences between the sleep and wake condition in children (*t*(190.6) = −1.59, *p* = 0.115, *p*_corr_ = 0.459); in adults the difference reached significance only on an uncorrected level (*t*(190.6) = 2.07, *p* = 0.04, *p*_corr_ = 0.161). Furthermore, the two age groups did not differ in performance in the wake condition (*t*(69.1) = 0.001, *p* > 0.999). However, children performed worse than adults in the sleep condition (*t*(69.1) = −2.68, *p* = 0.009, *p*_corr_ = 0.037, Figure 3b and Table S5). There were no further significant main effects or interactions (all *p* > 0.116).

### 3.2 Evaluative conditioning in a non‐social context

#### 3.2.1 Evaluative change

First, we checked for congruently paired Fribbles whether the evaluative conditioning worked. A significant interaction between valence and evaluation time (*F*(1,252) = 4.46, *p* = 0.036) showed that ratings for later positively and negatively paired Fribbles did not differ at baseline (*p* = 0.229), but showed a trend towards a more positive evaluation of positively paired Fribbles compared with negatively paired Fribbles (*p* = 0.09, *p*_corr_ = 0.364) at the delayed rating. While ratings did not change from baseline to delayed rating for positively paired Fribbles (*p* = 0.433), ratings decreased for negatively paired Fribbles (*p* = 0.035, *p*_corr_ = 0.141). Please note, none of the reported effects survived Bonferroni correction. There was no main effect of condition (*F*(1,252) = 1.62, *p* = 0.204), but a significant interaction between age group and valence (*F*(1,252) = 5.28, *p* = 0.022), with no significant pairwise comparisons. There were no further effects and interactions with respect to condition (*p >* 0.281; see Supplements Table S6 and Figure S4).

Since 17% of the trials represented Fribbles that were paired incongruently, we further analysed whether evaluations changed in the direction of the valence category to which the Fribbles belonged (rating in favour of the key information) or in the direction of the actual co‐presented UCS (item memory). There was a trend towards a significant interaction between evaluation time, valence category, and group (*F*(1,252) = 2.91, *p* = 0.089). Pairwise comparisons showed that children's ratings of Fribbles belonging to the positive valence category deteriorated from baseline (*M* = 62, *SEM* = 4.24) to delayed evaluation (*M* = 52, *SEM* = 4.25) indicating that these Fribbles were rated according to the actual co‐presented UCS (*p* = 0.025, *p*_corr_ = 0.099). Children's ratings did not change for Fribbles belonging to the negative valence category (*p* = 0.186). Adults' ratings did not change for any valence category (all *p* > 0.170, Figure S5).

#### 3.2.2 Feature generalisation

For the probability of correct decisions in the 2‐AFC task, we first tested against chance level (0.5) using two‐sided one‐sample Wilcoxon tests. Adults did not show below or above chance performance in any type of comparison (all *p* > 0.052). Children, however, showed above chance performance in the sleep condition, when a neutral species was paired with Fribbles from the negative category (*p* < 0.001, *p*_corr_ = 0.001), and below chance performance in both the sleep (*p* = 0.003, *p*_corr_ = 0.038) and wake condition (*p* = 0.035, *p*_corr_ = 0.42) when a neutral species was paired with a Fribble from the positive category.

**FIGURE 4:**
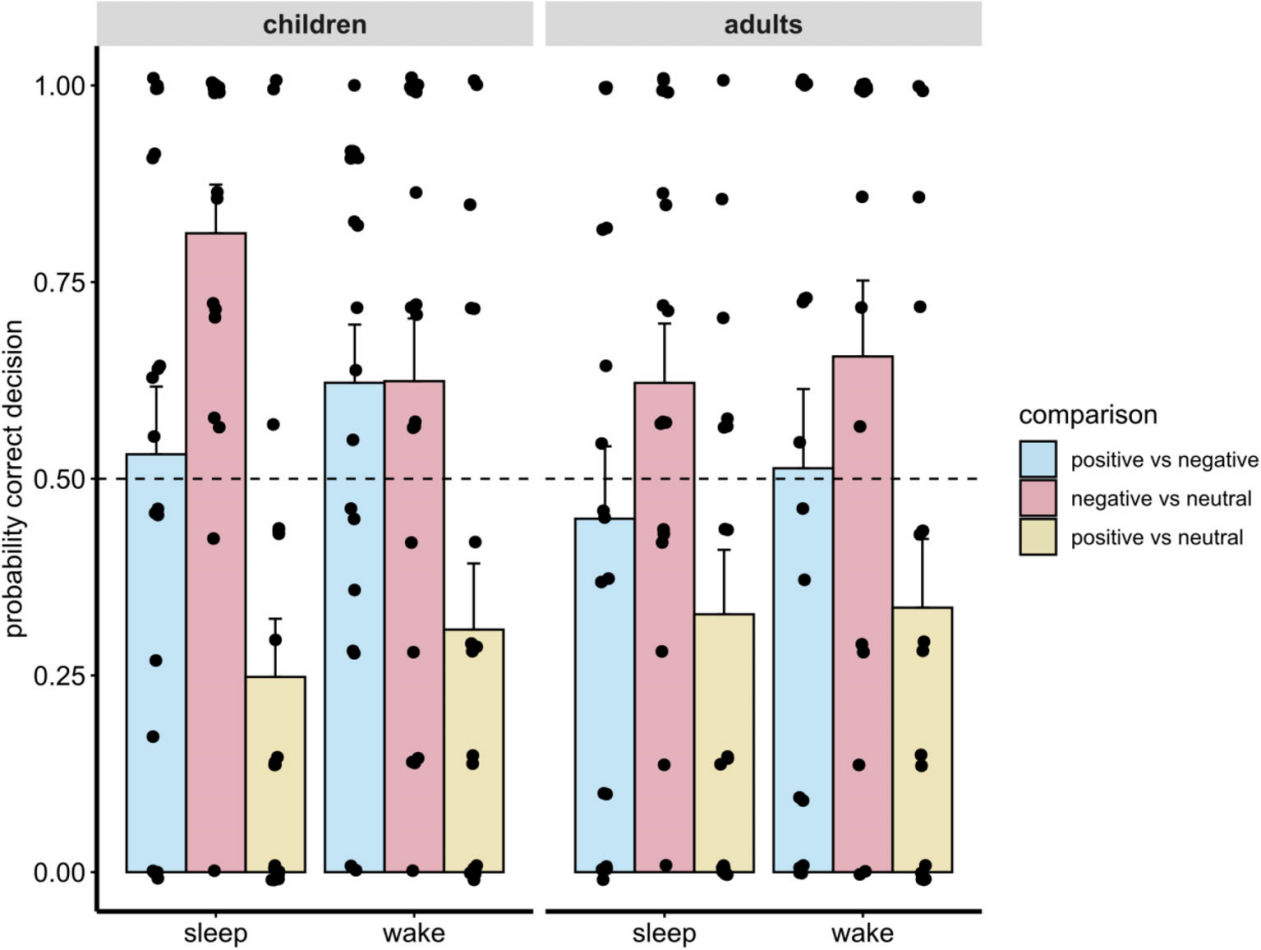
Evaluative conditioning task – feature generalisation: Probability of correctly deciding for the Fribble that belongs to the positive valence category (when simultaneously presented with a Fribble belonging to the negative valence category or a Fribble from an unknown species) or to an unknown species (when presented along a Fribble belonging to the negative valence category).

Subjecting group, condition, and type of comparison into one linear mixed‐effects model, showed only for the type of comparison a significant effect (*F*(2,180) = 26.94, *p* < 0.001). To our surprise, participants showed the worst performance when positive and neutral Fribbles (*M* = 0.30, *SEM* = 0.04) were presented simultaneously compared with positive vs. negative (*t*(191) = 4.25, *p*/*p*_corr_ < 0.001) or negative vs. neutral (*t*(191) = 7.09, *p*/*p*_corr_ < 0.001, Figure 4). When negative Fribbles were simultaneously presented with a neutral (*M* = 0.68, *SEM* = 0.04) or positive one (*M* = 0.53, *SEM* = 0.04), performance was better when presented alongside a neutral one (*t*(191) = −2.84, *p* = 0.005, *p*_corr_ = 0.015). There were no further main effects or interactions (all *p* > 0.281).

## 4 DISCUSSION

Here, we investigated whether sleep supports the generalisation of features that were extracted in a social and non‐social context in children and adults. For the social context, we found a sleep‐associated memory benefit in children and adults, whereas only adults benefit from sleep in terms of generalisation. Using an evaluative conditioning task, we did not find hints for a sleep‐benefit generalisation.

Considering that children need to adapt their behaviour to a complex environment by extracting relevant information from social and non‐social contexts, we investigated whether sleep supports this process more than in adults. For the social context, there was a sleep benefit in children and adults: while recognition performance remained stable across the interval filled with nighttime sleep, it deteriorated after daytime wakefulness. These data are in line with several studies showing that recognition performance is supported by sleep, which is true for pictures of items or for social visual stimuli (e.g. Kurz et al., 2019; Prehn‐Kristensen et al., 2017). Moreover, there was a trend that children showed better sleep‐associated memory performance compared with adults. In particular, our results closely resemble the findings of Wang et al. (2018), who similarly found a benefit of sleep for an episodic memory task across children and adults and additionally less forgetting in children than in adults. They further showed that children just forget less but showed worse memory performance 1 hour after encoding. Looking at the discrimination rates at the immediate recognition task instead of a change score in our task, we indeed also see worse immediate performance in children than adults. Nonetheless, children seem to show more effective consolidation than adults, even without the influence of sleep. While the type of offer (unfair, fair, friendly) did not appear to be relevant to adults in this regard, on exploratorion we observed that children benefit more from sleep only with respect to fair offers. Although our results should be discussed with care, the children's data suggest that the kind of social interaction with the proposer might influence later memory performance. In adults, Murty et al. (2016) observed that memory for offers depends on a social interaction with the proposer during encoding. In their study, however, memory was tested to the participant's surprise and only immediately after the encoding session. Extending those findings, our results suggest that sleep may amplify the memory for social offers – at least in children. Interestingly, sleep‐dependent memory performance in children was best when fair (in contrast to friendly and unfair) offers were made. One explanation for this result could lie in the selection of stimuli: There is evidence that children are better at recognising faces from their own age group compared with faces of adults (Hills & Lewis, 2011; Rhodes & Anastasi, 2012). Since in our study only adult faces were used as proposers, we assume that adults' faces who make fair offers appear more child‐oriented than adults' faces that make unfair or friendly offers. To reveal any possible interactions between the kind of offer and proposers' age, in an ongoing study we use pictures of proposers of the same age as the receivers.

For the main measure of interest, the ability to accurately predict the type of offer based on proposers' face features, we observed that both children and adults did not successfully extract the relevant features to predict the offer of an unknown face after wake. In contrast, after sleep, adults were better at predicting the type of offer than children. This result opposes our hypothesis that sleep benefits the extraction of implicitly learned rules more in children than in adults. On the other hand, there are studies showing that sleep in children does not support implicit memory as it does in adults (e.g. motor memory, odour memory, Prehn‐Kristensen et al., 2015; Wilhelm et al., 2008; Wilhelm et al., 2012). In those previous studies it was argued that children benefit from sleep only when certain levels of pre‐knowledge or stimulus familiarity are reached. Considering that sleep‐associated gaining insight into hidden rules relies on implicit memory performance (Verleger et al., 2013; Yordanova et al., 2008) we may have replicated previous findings on the sleep‐associated consolidation of implicit memory in children. From a biological perspective, it could also be argued that these kinds of rules only become established in children once they have proven themselves many times over. Against this background, favoured consolidation during sleep could even be a hindrance to stable development.

In the evaluative conditioning task, we did not observe a strong effect of conditioning in general: only the negative UCS changed the evaluation of the Fribbles after 12 hours but not the positive pictures. Based on our experiences with emotionally charged pictures (e.g. Prehn‐Kristensen et al., 2009, 2013), we carefully chose a set of positive and negative pictures expected to elicit sufficient emotions in children as well as in adults. Nonetheless, we cannot exclude that children and adults perceived the affective pictures differently leading to a bias during conditioning. Although evaluative conditioning in response to positive pictures did not differ between children and adults, it is known that the strongest positive arousal responses can be expected by erotic pictures (Jacob et al., 2011) – pictures that are not appropriate for children. From this point of view, the emotional strength of positive pictures might have been insufficient to change the evaluation after 12 h of retention.

To our surprise, we did not find any hints for a sleep benefit on the generalisation of information in the evaluative conditioning paradigm. This finding is at variance with our findings from the paradigm in a social context and with other studies showing better extraction of commonalities and generalisation after sleep compared with wakefulness (Graveline & Wamsley, 2017; Sandoval et al., 2017). It is conceivable that the effect of sleep on the reorganisation of some memory representations unfolds after some delay. Lutz et al. (2017) found an effect of sleep on the recognition of figural prototypes after 1 year only but not after 10 h. Yet, this seems not to be fully valid considering previous studies already found generalisation after very short delays using similar tasks (Halbeisen et al., 2017; Halbeisen et al., 2021). Since the evaluative condition has worked only for the negative pictures properly and given the overall small number of trials in the incongruent condition (*n* = 20), we refrained from analysing only negative conditioned Fribbles (*n* = 10).

Concerning the results of the evaluative feature generalisation (obtained from the 2‐AFC task) we observed that participants preferred the neutral Fribble in general, which was unexpected. However, since we asked: "Which of the two Fribbles do you like more?", the results might indicate that neutral (i.e. unknown) Fribbles were preferred to those that resemble the encoded ones. Perhaps the 120 repetitions of the same eight Fribbles during encoding caused participants to lose interest or to get bored with the Fribbles during retrieval that are very similar to those that had been encoded – independently of any positive or negative association. Instead, neutral (i.e. new) Fribble species might have been perceived as more interesting and therefore were "liked more". Maybe if we had asked "Which of the two Fribbles is associated with a positive picture?" the results might have changed in the predicted direction. If participants actually rated their preferences according to novelty, then the task resembled a recognition task (Frank & Kafkas, 2021); and interestingly on a descriptive level this process was mostly pronounced in the children in the sleep condition. However, this interpretation is highly speculative and needs to be confirmed in a follow‐up study first.

Due to several limitations, this study is not able to answer the question whether sleep benefits the abstraction of features from social and non‐social contexts in children more than in adults. One limitation is that in the social context, the faces of providers (adults) were not age‐matched to receivers (adults and children), which might have caused unexpected interactions between sleep and memory processes. In the evaluative conditioning task, a major issue was that the evaluation of the Fribbles did not properly change according to the emotional stimulus being associated with the Fribbles. Moreover, due to several repetitions during encoding, Fribbles might have become boring for the participants. Here, an introduction of a reward component might have increased the emotional engagement with the stimulus material as we observed in one former study (Wiesner et al., 2017).

Furthermore, successful feature abstraction was only implicitly assessed by generalisation. The decision not to ask the participants about any underlying regularities was grounded on the fact that we applied a within‐subjects design. Thus, we were unable to delineate whether generalisation was based on rule‐based or similarity‐based processes, that is whether participants were able to explicitly articulate the underlying rule or base their evaluation of new stimuli on the similarity to previously encoded items, respectively (e.g. Högden et al., 2020; Little & McDaniel, 2015).

Another limitation pertains to the age range of the children. Although we chose this age range to be comparable to previous studies finding better sleep‐dependent memory consolidation in children than adults (e.g. Peiffer et al., 2020; Prehn‐Kristensen et al., 2013; Wilhelm et al., 2013), we cannot exclude that the chosen age range was too heterogenous to be sufficiently distinct from adults. While the lower bound of 8 years was chosen to ensure the children understand the task instructions and can do both tasks in one session, the maximum of 12 years was based on the assumption that puberty is not yet at an advanced stage. Although markers of non‐REM sleep (delta activity) that are associated with brain maturation show their greatest decline after 12 years (Feinberg & Campbell, 2013), choosing smaller age ranges or further subdivisions of age groups are necessary to better understand developmental differences in sleep‐dependent memory processing. Furthermore, sleep recordings are required to prove an active role of sleep in gist abstraction.

Finally, the sample size of our study was too low to detect small effects. This was also confirmed by additional Bayesian analyses (not reported) that did not support any conclusions (neither supporting H0 nor H1) regarding the effect of age group and condition on the evaluative change of conditioned stimuli.

Taken together, we found signs of age‐dependent effects on the consolidation of quantitative and qualitative memory representations in a social context and to some extent also from evaluative conditioning. However, due to several limitations, we cannot draw final conclusions for the evaluative conditioning.

## AUTHOR CONTRIBUTIONS

**Eva‐Maria Kurz:** Writing – original draft; visualization; formal analysis. **Clara Marie Schreiber:** Conceptualization; writing – review and editing; methodology; software. **Konstantin Kölle:** Writing – review and editing; visualization. **Zeynep Tunçel:** Writing – review and editing. **Paula Theresa Meyer:** Writing – review and editing. **Hong‐Viet V. Ngo‐Dehning:** Writing – review and editing; funding acquisition. **Annette Conzelmann:** Writing – review and editing; funding acquisition. **Alexander Prehn‐Kristensen:** Conceptualization; writing – original draft; funding acquisition; methodology; writing – review and editing; resources.

## CONFLICT OF INTEREST STATEMENT

None.

## Supporting information


**Data S1.** Supporting Information.

## Data Availability

The data that support the findings of this study are available from the corresponding author upon reasonable request. The data are not publicly available due to privacy or ethical restrictions.
