# Supplementary material for: Does sleep help children to generalise features like adults?
Source: J Sleep Res. 2024 Dec 8;34(4):e14432. doi: 10.1111/jsr.14432 (PMC12215273; doi:10.1111/jsr.14432)
Supplement: Supplementary file 1 — Data S1. Supporting Information. [file JSR-34-e14432-s001.pdf]

Supporting Information

Tables

Table S1. Participant Characteristics

	children (n = 19)			adults (n = 17)		
	M	*SD*	range	*M*	*SD*	range
intelligence[1]	103.37	15.62	84-137	109.41	15.30	83-134
behavioral problems[2]	47.26	9.19	37-75	49.47	5.39	43-61
sleep[3]	41.05	5.85	37-75	4.71	3.00	1-12
	25.21	2.46	22-31			

Note. [1]Intelligence measured with either CFT-1-R (Weiß & Osterland, 2013) or CFT-20-R (Weiß, 2008). [2]Behavioral problems: global t-value from Child Behavior Checklist (Döpfner et al., 2014) for children; global severity index from the Symptom-Checklist-90 (Franke, 2014) for adults. [3]Total score from the Child Sleep Health Questionnaire (Schlarb et al., 2010, upper row) and Sleep-Self-Report (SSR, Schwerdtle et al., 2010, last row) for children and the Pittsburgh Sleep Quality Index (PSQI, Buysse et al., 1989) for adults. Seven children had a value >25 in the SSR and five adults exceeded the cut-off of 5 in the PSQI. Given the considerably high prevalence of sleep problems (30-40%) in children (Fricke-Oerkermann et al., 2007) and adults (Hinz et al., 2017) in the general population, these data reflect a realistic distribution of sleep problems in our study sample. Please note that no participant reported suffering from a diagnosed sleep disorder.

Table S2. Allocation of Fribbles to the parallel task versions based on category valence, and whether Fribbles were paired congruently or incongruently to the category valence.

Version	Valence category	Congruence			
		congruent		incongruent	
A	positive	Fb3_1313 (25x) 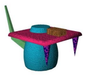	Fb3_3131 (25x) 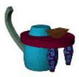	Fb3_1331 (5x) 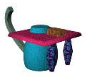	Fb3_3113 (5x) 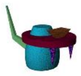
	negative	Fb4_1221 (25x) 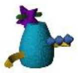	Fb4_2112 (25x) 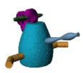	Fb4_1212 (5x) 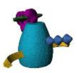	Fb4_2121 (5x) 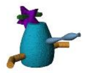
B	positive	Fc4_1313 (25x) 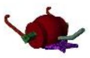	Fc4_3131 (25x) 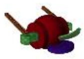	Fc4_1331 (5x) 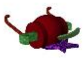	Fc4_3113 (5x) 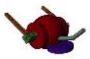
	negative	Fc2_1221 (25x) 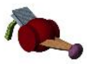	Fc2_2112 (25x) 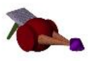	Fc2_1212 (5x) 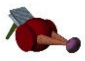	Fc2_2121 (5x) 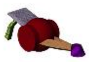

Note. Numbers in brackets indicate how often a Fribble was presented with the UCS, during the encoding session.

Table S3. Recognition of faces (d-prime) as the difference between delayed and immediate recognition

		children (n = 19)		adults (n = 17)	
		M	*SEM*	*M*	*SEM*
sleep	friendly	-0.07	0.22	0.23	0.30
	fair	0.40	0.18	-0.19	0.26
	unfair	0.21	0.28	-0.36	0.23
	combined	0.18	0.13	-0.10	0.15
wake	friendly	-0.22	0.16	-0.58	0.18
	fair	-0.47	0.23	-0.37	0.21
	unfair	-0.40	0.22	-0.77	0.24
	combined	-0.36	0.12	-0.57	0.12

Table S4. D-prime for the correct association of the type of offer for known (old) faces as the difference between delayed and immediate recognition

		children (n = 19)		adults (n = 17)	
		M	*SEM*	*M*	*SEM*
sleep	friendly	-0.20	0.23	-0.60	0.26
	fair	-0.36	0.23	-0.55	0.30
	unfair	-0.14	0.17	-0.36	0.18
	combined	-0.23	0.12	-0.50	0.14
wake	friendly	-0.38	0.23	-0.31	0.15
	fair	-0.44	0.21	-0.36	0.22
	unfair	-0.45	0.20	-0.31	0.18
	combined	-0.42	0.12	-0.33	0.10

Table S5. Prediction d-prime for the associated type of offer for new faces, as the difference between delayed and immediate recognition

		children (n = 19)		adults (n = 17)	
		M	*SEM*	*M*	*SEM*
sleep	friendly	-0.35	0.25	0.27	0.34
	fair	-0.26	0.17	0.47	0.31
	unfair	-0.37	0.13	0.17	0.21
	combined	-0.33	0.11	0.30	0.16
wake	friendly	-0.06	0.20	0.00	0.26
	fair	-0.06	0.23	-0.19	0.23
	unfair	-0.06	0.28	0.01	0.24
	combined	-0.06	0.13	-0.06	0.14

Table S6. Change in Fribble evaluation

		children (n = 19)				adults (n = 17)			
		sleep		wake		sleep		wake	
		M	*SEM*	*M*	*SEM*	*M*	*SEM*	*M*	*SEM*
congruent	positive	-1.66	6.69	-0.68	6.20	4.41	4.91	8.71	6.53
	negative	-6.71	4.75	-12.47	4.57	-0.27	4.29	-9.62	3.64
incongruent	positive	-9.71	6.60	-11.03	7.69	0.82	3.48	12.53	6.52
	negative	-4.05	5.03	-8.13	5.91	0.77	4.49	-10.21	3.51

Figures

Figure S1. Fribble composition.

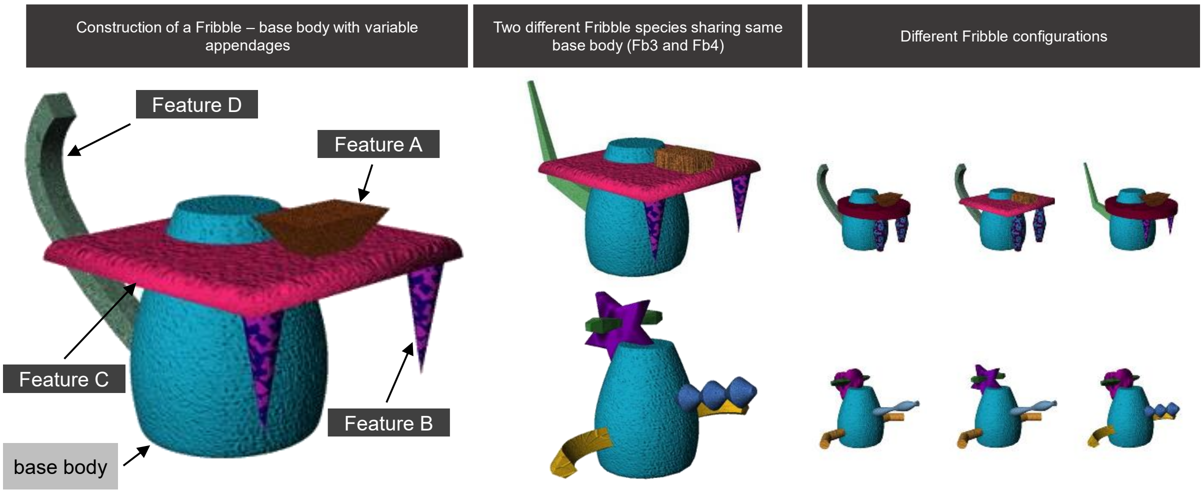

Note. Fribbles are constructs made up of different components, namely a base body and four additional appendages – Feature A to D. There are 12 different Fribble "species" with 4 species each sharing the same base body. Fribble species are differentiated by their distinct, species-specific configuration, shape, and color of appendages, so that differences between species are very marked and noticeable while differences within each species are rather granular in nature. The amount of possible combinations of appendages allows for 81 different Fribble configurations within each species. Due to the within-design of the current study, two species with different base bodies were used to present a new set of stimuli in each condition (sleep vs. wake). See Table S1 for an overview of all Fribbles that have been used in Version A and B.

Figure S2. Two-Alternative Forced Choice Task – Version A

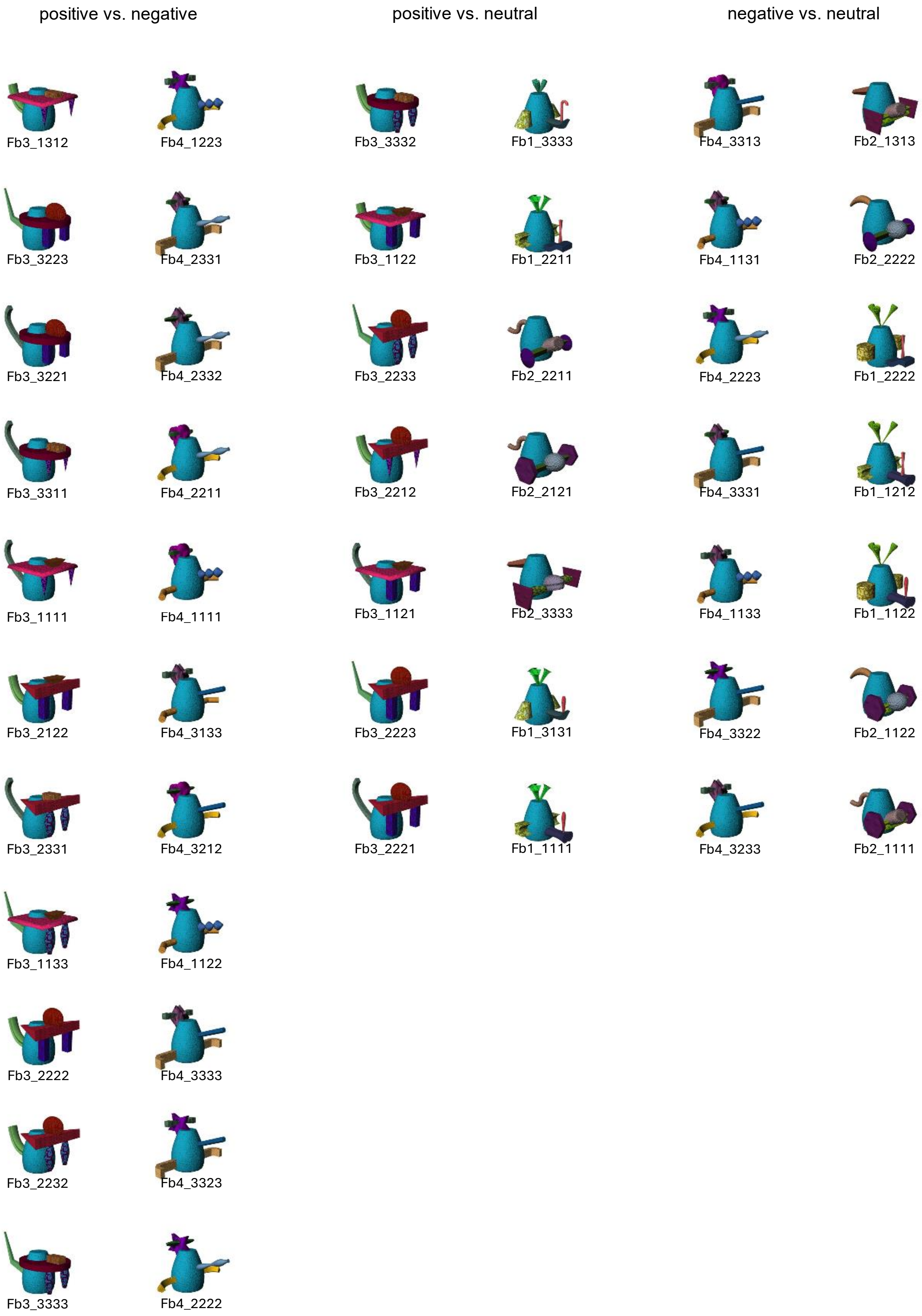

Figure S3. Two-Alternative Forced Choice Task – Version B

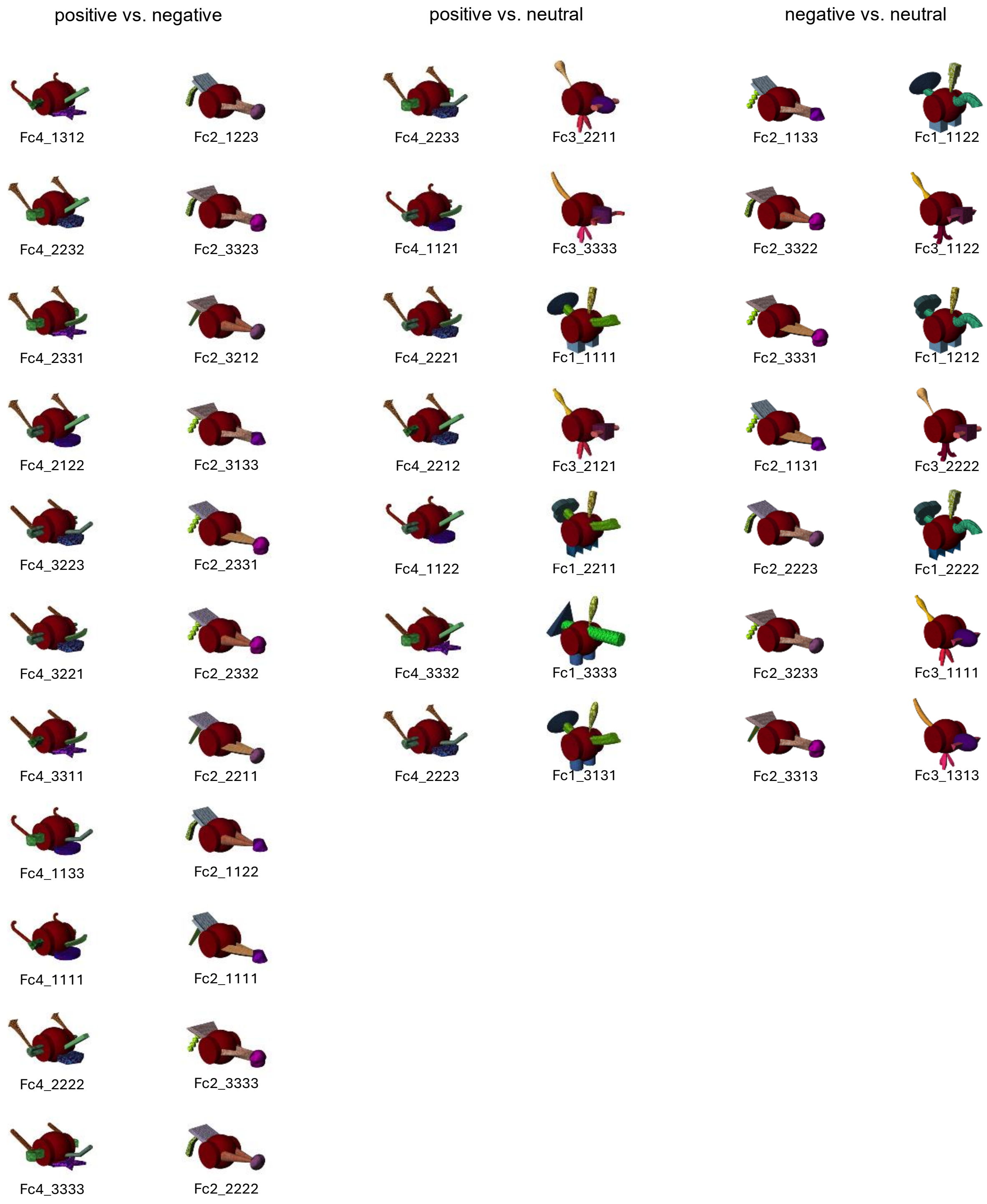

Figure S4. Change in evaluation for congruently paired Fribbles.

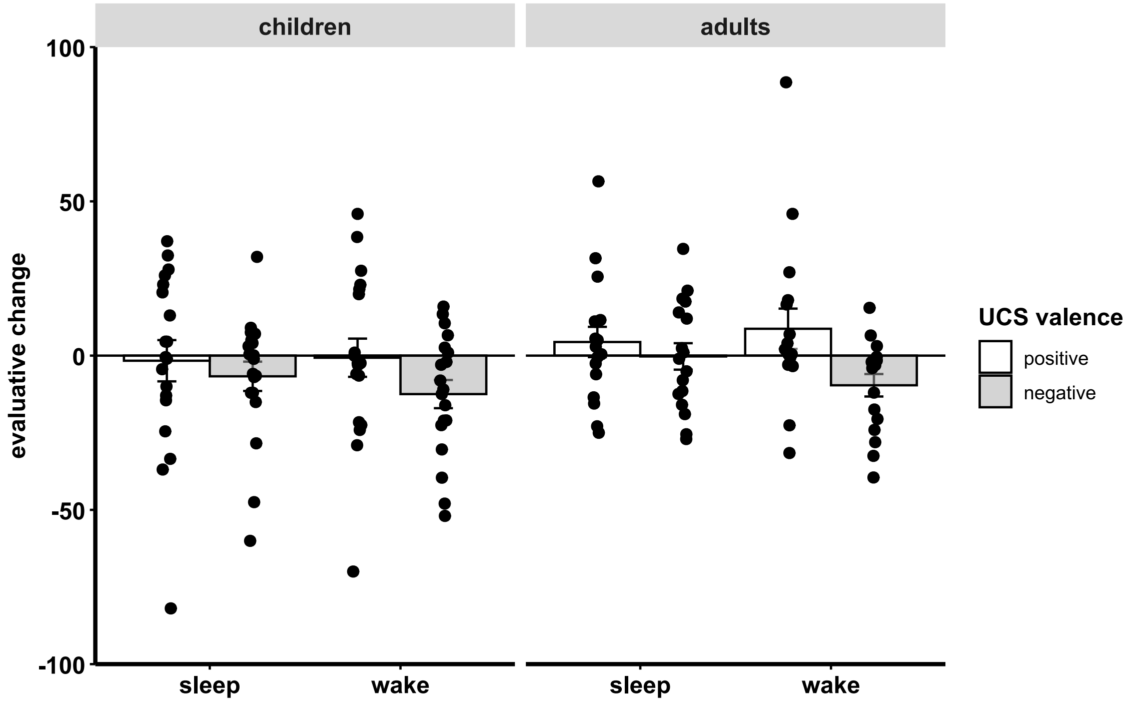

Figure S5. Change in evaluation for incongruently paired Fribbles.

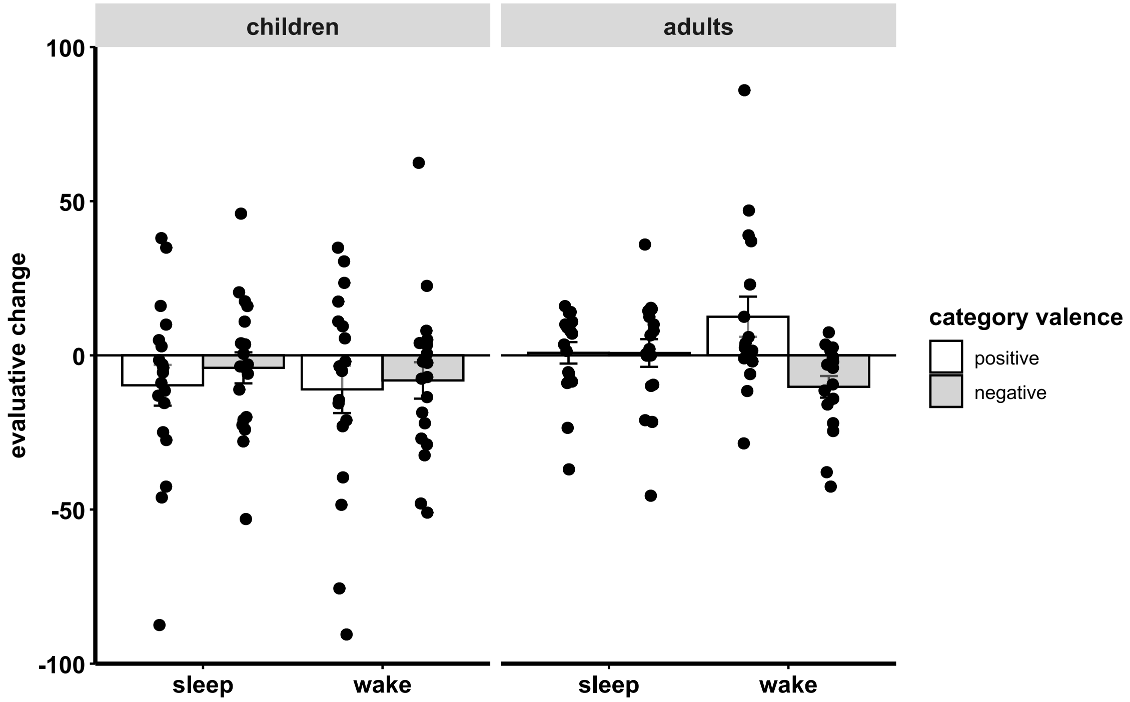

Note. Additionally to the effects described in the main text, we saw a marginally significant effect of condition (F(1,252)=3.77, p=.053) indicating generally less positive ratings in the sleep (M=48, SEM=1.93) compared to the wake condition (M=53, SEM=2.08).

Version balancing

In the reward-based feature generalization task in the social context, pictures for Version A and B were taken from the Chicago Face Database (Ma et al., 2015). To avoid any inferences between the proposer´s emotional face expression/ethnicity and the offer they made, we only selected pictures of people (all white) showing a neutral face expression. For these neutral pictures ratings are available at http://www.chicagofaces.org/.

In fact, picture sets were not counterbalanced according to sex (Set A: 53 females, Set B: 36 females). Please note that a complete equal distribution of males and females within Set A and Set B was not possible due to the carefully predefined facial features while the number of face pictures was limited. Most importantly however, based on the normative ratings (Ma et al., 2015), face expressions of Set A and B did not differ in terms of emotional expressions (afraid: p=.641, angry: p=.554, disgusted: p=.734, happy: p=.705, sad: p=.165, surprised: p=.248, threatening: p=.371, or trustworthy p=.916). Moreover, they did not differ in face shape (p=.942). We cannot exclude that the proposers' sex might have influenced the outcome to some degree; however, as we have endeavored to balance the allocation of the two versions across the sleep and wake condition, any possible differences between the sets can be assumed as controlled for.

Control variables

Control measures (alertness and sleepiness) were analyzed by means of linear mixed-effects models, with fixed effects for group (adults vs. children), condition (sleep vs. wake), session (encoding vs. retrieval), and, where applicable, assessment time (before vs. after performance of the memory tasks), and their interaction terms, as well as participants as random effect. Ratings of current levels of arousal, valence, and dominance were similarly evaluated using cumulative link mixed models (Christensen, 2015).

Means and standard errors for all control measures are reported in Table S7. Alertness levels were comparable in both conditions before encoding and retrieval (all p>.35). Merely a main effect of group indicated that adults (M=245ms, SD=26ms) reacted generally faster than children (M=343ms, SD=76ms; F(1,36)=34.16, p<.001).

Due to a significant three way interaction (group × condition × session, F(1,108)=9.69, p=.002), tiredness ratings were separately analyzed for children and adults. In children as well adults, we found a significant interaction between condition and session (all p<.031). While in adults the pairwise comparisons did not reach significance (all p>.11, all p_c >.45), children were significantly more tired at encoding (M=68, SEM=5.28) than retrieval (M = 52, SEM = 7.02), only in the wake condition (p = .04, p_c = 169). Importantly, there were no differences between the wake and sleep condition at encoding (p = .29, p_c > .999). However, at retrieval, children felt more tired in the wake than sleep condition (p = .053, p_c = .211). Adding the difference in tiredness between retrieval and encoding as covariate, did neither reveal any changes in results regarding our memory measures nor was the covariate itself significant. This was similarly the case, when sleepiness only at encoding was added as a covariate.

Valence ratings indicated better mood in the children than adults (main effect group: b = 1.71, 95% CI [0.29, 3.13], z = 2.37 p = .018) and generally better mood in the wake compared to the sleep condition (main effect condition: b = -0.47, 95% CI [-0.92, -0.03], z = -2.09, p =

.037), with no other significant effects (all $p > .07$). Probing any trends in interactions, did not reveal a difference prior encoding between the sleep and wake condition ($p = .73$).

Arousal ratings were higher in adults than children (main effect group: $b = 1.27$, 95% CI [0.25, 2.28], $z = 2.44$ $p = .015$). There was an interaction between session and assessment time ($b = 1.04$, 95% CI [0.18, 1.90], $z = 2.37$ $p = .018$), showing that arousal ratings were higher after retrieval than after encoding ($p = .03$, uncorrected). A trend in the interaction of condition × session × assessment time ($b = -1.66$, 95% CI [-3.37, 0.05], $z = -1.9$, $p = .057$) did not reveal a difference between the sleep and wake condition prior to encoding ($p = .76$).

Regarding dominance, no effects were found (all $p > .25$), except a trend ($b = -0.39$, 95% CI [-0.83, 0.05], $z = -0.53$, $p = .08$) towards an overall greater feeling of dominance in the sleep than the wake condition. To summarize, there are no hints of condition differences before encoding.

On a scale from 0 (not at all) to 100 (very), adults rated ($M = 54.35$ $SEM = 5.77$) their sleep between the encoding and retrieval session of the sleep condition less restorative than children ($M = 81.63$, $SEM = 5.77$; $F(1,34) = 14.26$, $p < .001$).

Table S7. Control Measures

		children (n = 19)				adults (n = 17)			
		sleep		wake		sleep		wake	
		M	*SEM*	*M*	*SEM*	*M*	*SEM*	*M*	*SEM*
Alertness	encoding	342.79	17.06	333.79	16.06	239.35	6.22	249.06	5.90
	retrieval	348.95	21.11	345.47	15.78	246.88	7.12	243.00	6.59
Sleepiness	encoding	59.53	6.44	68.00	5.28	59.94	4.93	49.59	5.44
	retrieval	67.21	6.04	51.58	7.02	49.71	4.02	59.53	5.41
Valence	pre encoding	2.74	0.37	2.79	0.35	3.59	0.43	3.53	0.26
	post encoding	2.95	0.39	2.37	0.33	3.77	0.33	3.65	0.31
	pre retrieval	2.74	0.34	2.42	0.35	3.82	0.27	3.12	0.38
	post retrieval	2.37	0.30	2.58	0.36	3.35	0.33	2.88	0.45
Arousal	pre encoding	3.21	0.40	3.47	0.39	3.88	0.48	3.77	0.35
	post encoding	2.74	0.33	2.84	0.41	3.12	0.34	4.00	0.42
	pre retrieval	2.68	0.39	3.05	0.39	3.59	0.39	4.29	0.38
	post retrieval	3.32	0.42	2.84	0.35	4.35	0.34	4.18	0.37

Dominance	pre encoding	6.74	0.50	6.21	0.48	6.06	0.42	5.65	0.40
	post encoding	6.84	0.49	6.79	0.38	6.12	0.36	5.71	0.39
	pre retrieval	6.47	0.52	6.42	0.49	6.24	0.38	5.71	0.38
	post retrieval	6.53	0.52	6.42	0.47	6.35	0.38	6.41	0.34

References

Buysse, D. J., Reynolds, C. F., 3rd, Monk, T. H., Berman, S. R., & Kupfer, D. J. (1989). The Pittsburgh Sleep Quality Index: a new instrument for psychiatric practice and research. *Psychiatry Res*, *28*(2), 193-213. https://doi.org/10.1016/0165-1781(89)90047-4

Christensen, R. H. B. (2015). ordinal—regression models for ordinal data. *R package version, 28*, 2015.

Döpfner, M., Plück, J., & Kinnen, C. (2014). *CBCL/6-18R-TRF/6-18R-YSR/11-18R: Deutsche Schulalter-Formen der Child Behavior Checklist von Thomas M. Achenbach: Elternfragebogen über das Verhalten von Kindern und Jugendlichen (CBCL/6-18R), Lehrerfragebogen über das Verhalten von Kindern und Jugendlichen (TRF/6-18R), Fragebogen für Jugendliche (YSR/11-18R)*. Hogrefe.

Franke, G. H. (2014). *SCL-90®-S: Symptom-Checklist-90®-Standard* (Vol. 1). Hogrefe.

Fricke-Oerkermann, L., Plück, J., Schredl, M., Heinz, K., Mitschke, A., Wiater, A., & Lehmkuhl, G. (2007). Prevalence and course of sleep problems in childhood. *Sleep*, *30*(10), 1371-1377. https://doi.org/10.1093/sleep/30.10.1371

Hinz, A., Glaesmer, H., Brähler, E., Löffler, M., Engel, C., Enzenbach, C., Hegerl, U., & Sander, C. (2017). Sleep quality in the general population: psychometric properties of the Pittsburgh Sleep Quality Index, derived from a German community sample of 9284 people. *Sleep Med*, *30*, 57-63. https://doi.org/10.1016/j.sleep.2016.03.008

Ma, D. S., Correll, J., & Wittenbrink, B. (2015). The Chicago face database: A free stimulus set of faces and norming data. *Behav Res Methods*, *47*(4), 1122-1135. https://doi.org/10.3758/s13428-014-0532-5

Schlarb, A. A., Schwerdtle, B., & Hautzinger, M. (2010). Validation and psychometric properties of the German version of the Children's Sleep Habits Questionnaire (CSHQ-DE). *Somnologie - Schlafforschung und Schlafmedizin*, *14*(4), 260-266. https://doi.org/10.1007/s11818-010-0495-4

Schwerdtle, B., Roeser, K., Kübler, A., & Schlarb, A. A. (2010). Validierung und psychometrische Eigenschaften der deutschen Version des Sleep Self Report (SSR-DE). *Somnologie - Schlafforschung und Schlafmedizin*, *14*(4), 267-274. https://doi.org/10.1007/s11818-010-0496-3

Weiß, R. H. (2008). *Basic Intelligence Test, Scale 2, CFT 20 (revised); Grundintelligenztest Skala 2 - Revision, CFT 20-R*. Hogrefe.

Weiß, R. H., & Osterland, J. (2013). *Basic Intelligence Test, Scale 1, CFT 1 (revised); Grundintelligenztest Skala 1 - Revision, CFT 1-R*. Hogrefe.
